# Comparison of the Intestinal Microbiota of Patients with Urticaria and Healthy Controls: The Role of Blastocystis

**DOI:** 10.3390/pathogens14111140

**Published:** 2025-11-11

**Authors:** Nurullah Ciftci, Salih Macin, Gülcan Saylam Kurtipek, Uğur Arslan

**Affiliations:** 1 Department of Medical Microbiology, Faculty of Medicine, Selcuk University, Konya 42130, Turkey; salihmacin@hotmail.com (S.M.); drarslanugur@gmail.com (U.A.); 2 Dermatology Department, Faculty of Medicine, Selcuk University, Konya 42130, Turkey; zeynep2011@selcuk.edu.tr

**Keywords:** *Blastocystis*, *Firmicutes*, intestinal microbiota, urticaria

## Abstract

Urticaria is a skin disorder characterized by erythematous, edematous, and pruritic lesions. Intestinal microorganisms can trigger various immunological responses, and Blastocystis has been suggested to affect gut-associated lymphoid tissue homeostasis and induce allergic reactions. This study aimed to evaluate the effect of Blastocystis on the intestinal microbiota in patients with urticaria. A total of 33 patients diagnosed with urticaria and 34 healthy controls were included. Independent sample *t*-tests, Welch’s *t*-tests, or Mann–Whitney U tests were applied to assess differences in the Shannon, Simpson, and Chao-1 indices between groups. Significant differences were observed in Proteobacteria (*p* = 0.015), Bacteroidetes (*p* = 0.008), Escherichia (*p* = 0.005), Phocaelcola (*p* = 0.043), and Prevotella (*p* = 0.047) between the urticaria and control groups. Bacteroidetes (*p* = 0.003) and Phocaelcola (*p* = 0.032) also differed significantly between samples with and without Blastocystis. Overall microbiota composition showed a significant difference between Blastocystis-positive and -negative samples (*p* = 0.009). The Firmicutes/Bacteroidetes ratio was 4.1 in healthy controls and 6.4 in urticaria patients. In conclusion, both urticaria and Blastocystis infection significantly influence intestinal microbiota composition, suggesting a potential interaction between Blastocystis colonization and host immune regulation in urticaria.

## 1. Introduction

Urticaria is a skin disorder characterized by itchy, edematous papules or plaques, and sometimes angioedema resulting from the involvement of the deep dermis and subcutis. When the duration of symptoms is less than six weeks, it is defined as acute urticaria, whereas persistence for six weeks or longer is classified as chronic urticaria. Chronic urticaria significantly reduces patients’ quality of life and poses therapeutic challenges for clinicians. Although the etiology of chronic urticaria remains uncertain, its pathogenesis is thought to involve dysregulation of coagulation, inflammatory, and immune pathways [1].

*Blastocystis* spp. are regarded as a common component of the human intestinal microbiota, although their pathogenic potential remains controversial. There are approximately 17 subtypes of *Blastocystis* spp., but only nine of them have been detected in humans. Some subspecies have been shown to be related with pathogenicity [2]. *Blastocystis hominis* is known to influence the homeostasis of gut-associated lymphoid tissue and may trigger allergic reactions through its antigens or direct host interaction. Identifying the associations between Blastocystis subtypes and various diseases is crucial for improving diagnostic accuracy and guiding appropriate treatment strategies [2,3].

In recent years, microbiota and microbiome research has gained considerable importance in the field of microbiology. Such studies primarily aim to elucidate the complex interactions between microorganisms and their hosts. Although bacteria constitute the majority of the human microbiome, it also comprises viruses, fungi, and other eukaryotic microorganisms that collectively contribute to host physiology and immune regulation [4,5]. The human genome is estimated to be approximately 150 times smaller than the collective bacterial genome within the human body. Similarly, the number of microbial cells in humans is estimated to exceed the number of human cells by about tenfold [4,6]. The human microbiota is primarily located in the digestive tract, skin, genitourinary, and respiratory systems. More than 70% of the microorganisms in the human body reside in the colon. The gut microbiome comprises diverse bacterial phyla, with Firmicutes, Bacteroidetes, Actinobacteria, and Proteobacteria being the most prevalent. Among these, Firmicutes accounts for approximately 79.4% of the gut microbiome, followed by Bacteroidetes at 16.9%. The density of microorganisms increases progressively from the proximal to the distal gastrointestinal tract and from the epithelial surface toward the lumen. This gradient is attributed to the harsh environment of the upper intestines, where gastric acid, digestive enzymes, and rapid chyme transit limit microbial colonization, while the lower intestines provide reduced oxygen tension favorable to facultative and obligate anaerobes [7,8,9]. 

Recent studies suggest that the human microbiome plays a critical role in both health and disease, and has therefore been described as an overlooked organ. The microbiota contributes to the development and maturation of immune system cells and provides essential signals that regulate immune responses and influence host susceptibility to various diseases [10,11,12]. In this study, we investigated the association between the intestinal microbiota and urticaria. Specifically, we aimed to examine differences in the gut microbiota of patients who tested positive for *Blastocystis* spp. and to determine the potential role of Blastocystis in urticaria. There is limited clinical research in Turkey examining the relationship between parasitic infections and urticaria. Therefore, this study aims to elucidate the influence of *Blastocystis* spp. on the intestinal microbiota of urticaria patients, providing valuable insights for diagnosis and treatment, as well as important epidemiological contributions.

## 2. Materials and Methods

Between 1 January and 31 December 2021, a total of 67 stool samples were analyzed in the Medical Microbiology Laboratory. Of these, 33 samples were obtained from patients who visited the dermatology clinic with urticaria complaints. For comparison, 34 stool samples were collected from age- and sex-matched healthy individuals. Participants who had used antibiotics within three months prior to sampling were excluded from the study.

The study population was divided into four groups:

Urticaria patients with *Blastocystis* spp. detected in stool (n = 19).

Urticaria patients without *Blastocystis* spp. detected in stool (n = 14).

Healthy individuals with *Blastocystis* spp. detected in stool (n = 16).

Healthy individuals without *Blastocystis* spp. detected in stool (n = 18).

Upon arrival at the laboratory, stool samples were examined for *Blastocystis* spp. under a light microscope at 40× magnification and then stored at −20 °C until molecular analyses. Samples from healthy individuals were also microscopically examined, and suitable specimens were stored for analysis. All *Blastocystis*-positive samples were confirmed by PCR before inclusion in the study.

### 2.1. DNA Extraction

Approximately 200 ± 20 mg of each stool sample was used for DNA extraction following the manufacturer’s instructions, using the ZymoBIOMICS DNA Stool Miniprep Kit (Zymo Research, Tustin, CA, USA, 2021). The concentration of extracted DNA was measured using a biophotometer (Eppendorf AG, Hamburg, Germany). The purity of DNA was evaluated by spectrophotometry, and all DNA samples were stored at −80 °C until further analysis.

### 2.2. PCR

DNA amplification was performed using the following primers:

Forward primer (23 bp): 5′-GGTCCGGTGAACACTTTGGATTT-3′.

Reverse primer (24 bp): 5′-CCTACGGAAACCTTGTTACGACTA-3′.

PCR conditions consisted of an initial denaturation at 95 °C for 2 min, followed by 35 cycles of denaturation at 95 °C for 30 s, annealing at 58 °C for 30 s, and extension at 72 °C for 35 s, with a final extension at 72 °C for 7 min. The PCR protocol used in this study was based on the procedure recommended by Oxford Nanopore Technologies, Oxford, UK (2021).

The reaction mixture (final volume 50 μL) contained:

5 μL of 10× Taq buffer, 1 μL of dNTPs (10 mM), 5 μL of DNA (1 μg/μL), 1 μL of forward primer, 1 μL of reverse primer (10 pmol), 4 μL of MgCl_2_ (25 mM), 0.3 μL of Taq polymerase, and 32.7 μL of sterile distilled water.

**Figure 1:**
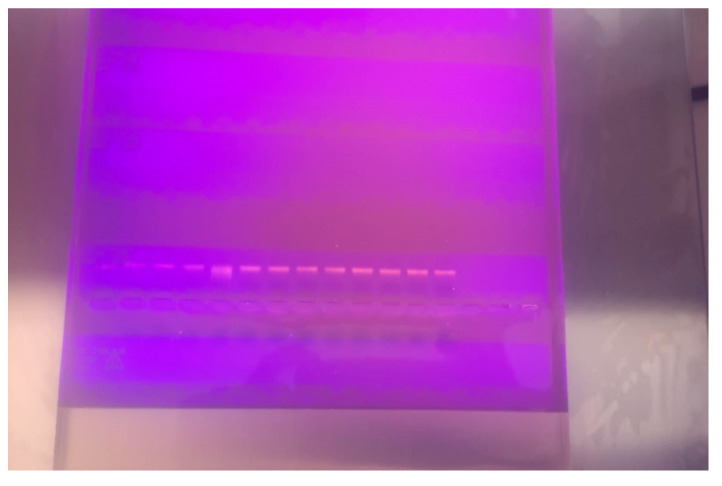
Execution of Nucleic acids isolated from patient and healthy groups by Agarose Gel Electrophoresis.

PCR products were separated by electrophoresis on a 2% agarose gel containing ethidium bromide, alongside a 200 bp DNA ladder (Promega, San Luis Obispo, CA, USA). Specific bands were visualized using a transilluminator under UV light (Gel Logic 200 Imaging System, Kodak, Rochester, NY, USA), and restriction fragments of each isolate were analyzed (Figure 1).

### 2.3. 16s rRNA Sequencing

Sequencing was performed according to the manufacturer’s (Oxford Nanopore Technologies, UK, manufactured in 2021) recommendations as follows:

1. Amplification of the 1500 bp 16S rRNA region using tailed universal primers (27F and 1492R) with LongAmp Taq 2× Master Mix (NEB M0287) (Oxford Nanopore Technologies, Oxford, UK).
2. Purification of the 1500 bp amplicon and quantification of DNA concentration.
3. Barcoding of each sample using the PCR Barcoding Expansion Pack 1–96 (EXPPBC096) protocol (Oxford Nanopore Technologies, Oxford, UK).
4. DNA end repair and adapter preparation using the LSK109 1D Ligation Kit (Oxford Nanopore Technologies, Oxford, UK).
5. Adapter ligation with the NEBNext Quick T4 DNA Ligase (SQK-LSK109, Oxford Nanopore Technologies, Oxford, UK).
6. Purification of the ligated products.
7. Flow cell priming and sample loading.
8. Sequencing initiation using MinKNOW V.5.2.3. software, Oxford Nanopore Technologies, Oxford, UK).

### 2.4. Bioinformatic Analysis

Comparative metagenomic analyses were conducted to examine the bacterial composition of the 67 samples. Relationships between metadata categories and bacterial community profiles were assessed using several analytical methods, including Krona visualization, genus-based analyses, similarity analyses, alpha diversity indices, PCA, beta diversity analyses, and LEfSe analysis.

### 2.5. Statistical Data

All statistical analyses were performed using R version 3.6.0 (The R Foundation for Statistical Computing, Vienna, Austria; https://www.r-project.org, accessed on 28 February 2020). The Shapiro–Wilk test and Q–Q plots were used to assess data normality, and Levene’s test was applied to evaluate homogeneity of variances. Numerical data are presented as mean ± standard deviation (range: min–max) or as median and interquartile range (IQR; 25th–75th percentile). The Mann–Whitney U test was used to determine statistically significant differences in Shannon, Simpson, and Chao-1 indices between study groups. A two-tailed *p*-value < 0.05 was considered statistically significant.

## 3. Results

A total of 33 samples were obtained from patients diagnosed with urticaria (with or without *Blastocystis* spp.) and 34 from healthy controls. The control group included 16 males and 18 females, while the urticaria group included 16 males and 17 females. The mean age was 29 years in the control group and 41 years in the patient group, showing a statistically significant difference (*p* < 0.05). No significant age difference was found between groups with or without *Blastocystis* spp.

### 3.1. DNA Isolation Results

DNA concentrations were measured using a Nanodrop spectrophotometer (Maestrogen, Hsinchu, Taiwan). Samples with low DNA concentrations were re-extracted. Prior to sequencing, all nucleic acid isolates were evaluated by agarose gel electrophoresis to confirm band presence (Figure 1). Final nucleic acid concentrations were quantified using a Quantus Qubit Fluorometer (Promega, Lyon, France) before initiating sequencing.

### 3.2. Comparison of Study Groups According to Shannon, Simpson, and Chao-1 Indices

The alpha diversity indices (Shannon, Simpson, and Chao-1) were calculated to assess the richness and evenness of bacterial communities in the intestinal microbiota of all study groups. The mean values of these indices are presented in Table 1.

**Figure 2:**
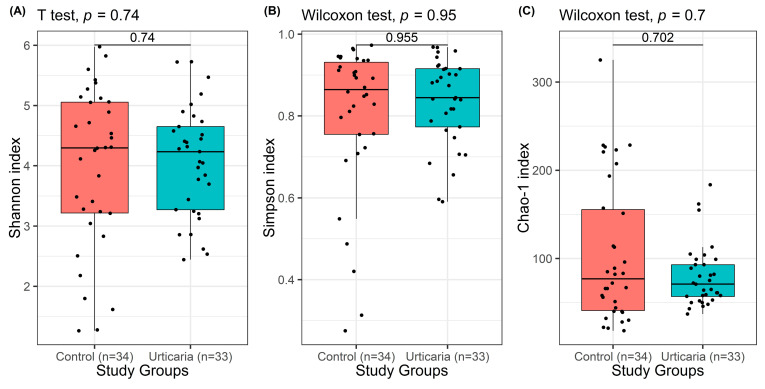
Box plots showing the Shannon (**A**), Simpson (**B**), and Chao-1 (**C**) indices in patients with urticaria and healthy controls. Horizontal lines within each box indicate the median. Data are presented as medians with interquartile ranges (IQR). The Welch’s *t*-test and Mann–Whitney *U* test (unpaired Wilcoxon test) were used for comparisons between healthy controls and patients with urticaria.

When comparing patients with urticaria to healthy controls, no statistically significant differences were observed in any of the diversity indices (Shannon: *p* = 0.740; Simpson: *p* = 0.955; Chao-1: *p* = 0.702) (Figure 2). These findings indicate that the overall microbial diversity and richness of bacterial taxa in patients with urticaria were similar to those in healthy individuals. Although the diversity indices did not differ significantly between groups, the data showed a slightly higher variability in the urticaria group compared to controls, suggesting subtle shifts in microbial composition rather than a general loss or gain in diversity. This finding may indicate that urticaria, as a multifactorial disease, is associated more with qualitative alterations in specific bacterial taxa rather than quantitative changes in total microbial diversity.

### 3.3. Comparison of Study Groups According to the Presence of Blastocystis spp.

**Figure 3:**
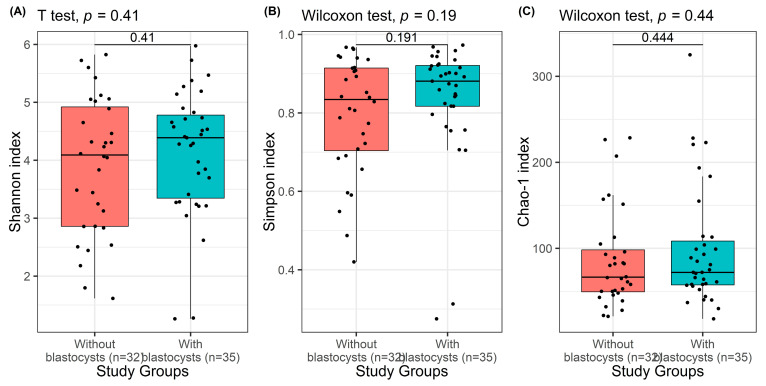
The box-plot of Shannon (**A**), Simpson (**B**), and Chao-1 (**C**) index in individuals with and without *Blastocystis* spp. Horizontal lines in each box indicate the median. Data were represented as median with interquartile ranges. The Independent sample *t*-test and Mann–Whitney *U* test (Unpaired Wilcoxon test) were used for comparison among individuals with and without *Blastocystis* spp.

The alpha diversity indices (Shannon, Simpson, and Chao-1) for all study groups are presented in Table 2 and Figure 3. These indices were used to evaluate the richness and evenness of bacterial taxa within the intestinal microbiota of individuals with and without *Blastocystis* spp. When comparing *Blastocystis*-positive and -negative individuals, no statistically significant differences were observed in the Shannon, Simpson, or Chao-1 indices (*p* = 0.408, *p* = 0.191, and *p* = 0.444, respectively). This finding indicates that the overall bacterial diversity and richness in the intestinal microbiota were not substantially influenced by the presence or absence of *Blastocystis* spp.

Although the alpha diversity values were similar across groups, a slightly wider distribution in the *Blastocystis*-positive samples was observed, suggesting a more heterogeneous bacterial composition among these individuals. This may reflect potential strain-level or subtype-related variability of *Blastocystis* spp., which could exert subtle effects on specific bacterial taxa rather than causing a generalized alteration in total microbial diversity. Overall, these results suggest that Blastocystis colonization does not significantly alter the overall richness or evenness of the gut microbiota but might be associated with compositional differences at specific taxonomic levels, which warrant further investigation.

### 3.4. Displaying Microbial Community Diversity and Quantity Graphs of Samples at Phylum Level in Percentage (Level 2 and Level 6)

**Figure 4:**
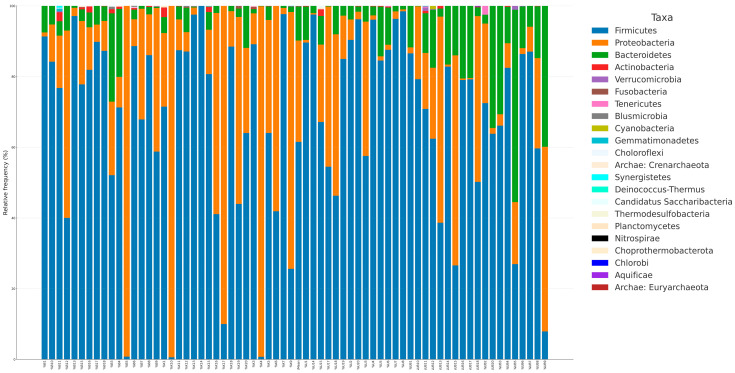
Diversity in microbial community at the phylum level (Level 2). B: *Blastocycstis* spp.-positive, urticaria-negative; K: *Blastocycstis* spp.-negative, urticaria-negative (Healthy Control); U: Urticaria-positive, *Blastocycstis* spp.-Negative; UB: Urticaria-positive, *Blastocycstis* spp.-positive.

**Figure 5:**
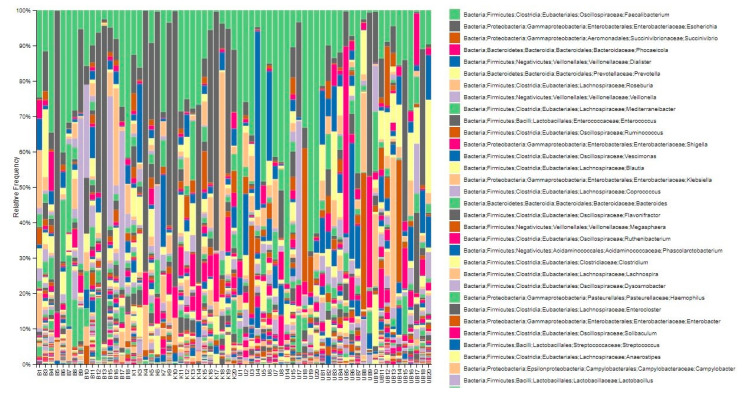
Microbiota analyses of samples at the genus level (Level 6). B: *Blastocycstis* spp.-positive, urticaria-negative; K: *Blastocycstis* spp.-negative, urticaria-negative (Healthy Control); U: Urticaria-positive, *Blastocycstis* spp.-Negative; UB: Urticaria-positive, *Blastocycstis* spp.-Negative.

In the microbiota analysis, taxonomic profiling of the bacterial community was performed to evaluate the relative abundance and diversity of taxa across samples. The compositional distribution of the microbiota was visualized at two different taxonomic levels: phylum (Level 6) and genus (Level 2) (Figure 4 and Figure 5).

Bar charts were generated to display the proportion of bacterial taxa within each sample, expressed as percentages. These visualizations allow for a comparative assessment of the dominant phyla and genera between the study groups. The stacked bar charts in Figure 4 (phylum level) and Figure 5 (genus level) illustrate the most prevalent microbial taxa identified in each sample and highlight the variability in microbial composition among individuals.

Overall, these graphical representations provide an overview of the relative abundance of the most frequently detected microorganisms, enabling the identification of compositional differences that may be associated with urticaria or the presence of *Blastocystis* spp.

### 3.5. Dendrogram Based on the Diversity of Samples

**Figure 6:**
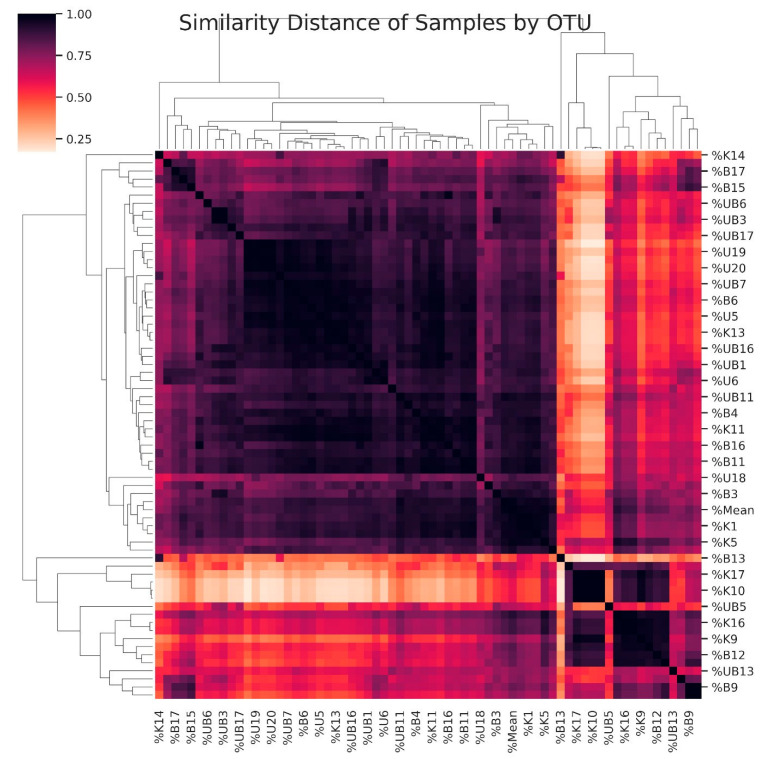
Clonal similarity difference among samples by Operational Taxonomic Unit (OTU).

A dendrogram was constructed to visualize the similarity relationships among all samples based on their microbial community composition (Figure 6). In the dendrogram, similarities between samples are represented using a color-coded matrix on both the horizontal and vertical axes, with similarity values ranging from 0 to 1. As the similarity coefficient increases (approaching 1), the color intensity becomes darker, indicating a higher degree of similarity between samples. Conversely, lighter colors represent lower similarity values (approaching 0), reflecting greater dissimilarity in microbial composition. This analysis considered all taxonomic levels, thereby capturing both qualitative (presence or absence) and quantitative (relative abundance) aspects of microbial diversity. The resulting dendrogram illustrates how samples luster according to their microbial community structure, providing insights into the overall relationships among study groups.

### 3.6. Microbiota Diversity Between Control and Urticaria Patients

In this study, microbiota analyses were conducted on 33 patients with urticaria and 34 healthy controls. The bacterial taxa detected in both groups were statistically evaluated using the Mann–Whitney U test. Significant differences were observed in the relative abundance of several bacterial taxa between the two groups, including Proteobacteria (*p* = 0.015), Bacteroidetes (*p* = 0.008), Escherichia (*p* = 0.005), Phocaelcola (*p* = 0.043), and Prevotella (*p* = 0.047) (Table 3). When the overall bacterial composition of the microbiota was analyzed, a statistically significant difference was also detected between urticaria patients and healthy controls (*p* = 0.026). These findings suggest that individuals with urticaria exhibit notable alterations in their intestinal microbiota compared to healthy individuals.

In our study, the Firmicutes/Bacteroidetes (F/B) ratio was determined to be 4.1 in healthy controls and 6.4 in patients with urticaria. Although the F/B ratio was higher in urticaria patients compared to healthy individuals, this difference was not statistically significant (*p* = 0.22). These results indicate that both urticaria and *Blastocystis* infection have a notable impact on the composition of the intestinal microbiota.

### 3.7. Microbiota Differences Between Blastocystis-Positive and -Negative Samples

In this study, microbiota analyses were performed on 32 Blastocystis-negative and 35 Blastocystis-positive samples at both the phylum (level 6) and genus (level 2) levels. Statistical comparisons between the two groups were conducted using the Mann–Whitney U test. Significant differences were observed in the abundance of *Bacteroidetes* (*p* = 0.003) and *Phocaelcola* (*p* = 0.032) between *Blastocystis*-positive and -negative samples. Both bacterial taxa also showed statistically significant differences within the urticaria subgroups. Moreover, when the overall microbiota composition of *Blastocystis*-positive and -negative samples was compared, a statistically significant difference was detected (*p* = 0.009) (Table 4).

## 4. Discussion

Previous studies have demonstrated that individuals infected with *Blastocystis* may exhibit accompanying urticaria symptoms. This phenomenon is thought to result from immunological reactions triggered by pathogenic microorganisms residing in the intestinal system. It has been reported that *Blastocystis*, either through its antigens or by direct interaction, can influence the homeostasis of intestinal-associated lymphoid tissues and consequently induce allergic or hypersensitivity reactions [13]. *Blastocystis* activates inflammatory pathways in host immune system cells, especially macrophages, and can induce some cytokines (IL-1β, IL-6,9 and tumor necrosis factor-alpha (TNF-α) [14]. Increased interleukin levels and Toll-Like Receptor (TLR) regulation have been demonstrated using specific ligands in IBS patients with *Blastocystis* spp. Infection [15,16]. A recent study has emphasized that Blastocystis subtypes can interact differently with the gut microbiota, potentially altering the balance between commensal and pathogenic microorganisms. In the study postbiotic compounds derived from *Pediococcus acidilactici* exhibited antiprotozoal activity against *Blastocystis* subtypes ST1 and ST3, suggesting that modulation of the gut microbiota through postbiotic interventions could offer a promising therapeutic strategy for *Blastocystis*-associated dysbiosis [17].

Microbiota dysbiosis can lead to dysregulation of bodily functions and diseases including cardiovascular diseases (CVDs), cancers, respiratory diseases, allergic diseases, urticaria, etc. [18,19,20]. In healthy conditions, the gut microbiota exhibits stability, resilience, and symbiotic interaction with the host. There is a lot of research into the definition of a “healthy” gut microbiota and its link to host physiological functions [21]. In a healthy person, the intestinal microbiota mainly comprises Gram-positive bacteria (*Firmicutes* and *Actinobacteria*) and Gram-negative bacteria (Bacteroidetes and Proteobacteria). Studies show that the rate of *Firmicutes* to *Bacteroides* (F/B) has a significant relationship with the composition of the human intestinal microbiome, making it an important parameter reflecting the intestinal microbiota composition [22,23,24]. In a comparative study of the gut microbiome across three age groups (infants, adults, and the elderly), the F/B ratio was found to be 0.4, 10.9, and 0.6, respectively. A high difference in F/B ratio was observed between the infant and adult age groups, but no significant difference was detected between the infant and elderly groups. In this study, researchers found that the F/B ratio increased from birth to adulthood, but then started to decrease again at older ages. It has been reported that the reason for this microbiota difference is the differences in daily nutritional needs in people depending on age [22]. In our study, the Firmicutes/Bacteroidetes (F/B) ratio was determined to be 4.1 in healthy controls and 6.4 in patients with urticaria. Although the F/B ratio was higher in urticaria patients compared to healthy individuals, this difference was not statistically significant (*p* = 0.22). This finding suggests that urticaria may influence the F/B ratio, which is considered an important indicator of the overall status of the human intestinal microbiome. However, the literature does not provide definitive reference values for the F/B ratio in healthy individuals. It appears that both markedly increased and decreased F/B ratios are associated with various pathological conditions [25].

The composition and function of the gut microbiota in Blastocystis-colonized individuals can be influenced by several external factors, including diet quality and socioeconomic status. In a study suggest that dietary habits significantly modulate the gut microbial structure in individuals colonized by Blastocystis, suggesting that host lifestyle and nutrition may determine whether the presence of Blastocystis results in dysbiosis or remains asymptomatic [26]. Recent experimental evidence supports the idea that Blastocystis colonization can lead to measurable changes in gut bacterial communities. For example, in vitro and in vivo studies using subtype ST7 demonstrated that Blastocystis infection resulted in a significant reduction in beneficial genera such as *Bifidobacterium* longum and *Lactobacillus* spp., while allowing for increase in potentially opportunistic taxa such as *Escherichia coli* [27]. These findings parallel our observations of decreased abundance in Bifidobacteriaceae and increased *Escherichia* in urticaria patients, suggesting that Blastocystis-mediated dysbiosis may contribute to immune-mediated skin disease. Another large scale human study found that Blastocystis carriage was significantly associated with a lowered Firmicutes/Bacteroidetes ratio (F/B ratio) in both healthy and metabolically ill populations [28]. While our study found a higher F/B ratio in urticaria patients (6.4 vs. 4.1 in controls), lack of statistical significance (*p* = 0.22) may reflect different disease context, microbial community dynamics, or subtype distribution. Nonetheless, the direction of change warrants further investigation, especially in skin immune-mediated disorders.

Metagenomic DNA analysis revealed significant differences in bacterial composition between patients with urticaria and healthy controls. Specifically, members of the *Bacteroidetes* phylum, as well as the families *Lachnospiraceae*, *Ruminococcaceae*, and *Clostridiaceae*, and the genera *Intestinibacter*, *Megasphaera*, and *Sutterella*, were found to be significantly increased in patients with urticaria. Conversely, a significant decrease was observed in bacterial groups belonging to the families *Bifidobacteriaceae*, *Lachnospiraceae*, *Ruminococcaceae*, *Veillonellaceae*, *Prevotellaceae*, and *Coriobacteriaceae*, as well as in members of the order *Clostridiales* and the genus *Succinivibrio*. These alterations in microbial composition may be associated with the clinical course of chronic urticaria and may reflect dysbiotic mechanisms contributing to disease pathogenesis.

A limitation of this study is the relatively small sample size and the lack of subtype analysis of *Blastocystis* spp., as different subtypes may vary in their pathogenic potential. Age difference between the patient and control groups, which may have influenced the gut microbiota composition, is another limitation of the study. However, this difference reflects the real-world demographic profile of patients with urticaria, and no significant age difference was found between subgroups with or without *Blastocystis* spp. Therefore, the influence of age on the main findings is likely limited. The evaluation of Blastocystis was conducted using microscopy and PCR, which confirm the presence of the organism but do not provide information regarding its subtypes. It is necessary to consider that the gut microbiota can vary regionally (the sample was entirely from Turkey).

## 5. Conclusions

Differences between bacterial communities in the intestinal microbiota suggest that there is a significant relationship between chronic urticaria disease and intestinal dysbiosis. These alterations in microbial composition may be associated with the clinical course of chronic urticaria and may reflect dysbiotic mechanisms contributing to disease pathogenesis. Our findings support the hypothesis that disruption of the intestinal microbiota may play a key role in the immunopathogenesis of urticaria. Moreover, this study provides one of the few datasets from Turkey investigating the relationship between *Blastocystis* spp. infection and gut microbiota composition in urticaria patients. Therefore, it contributes valuable data to the understanding of microbiota-related immune mechanisms and highlights the importance of considering intestinal parasites, particularly Blastocystis, in the clinical evaluation of chronic urticaria. Therefore, creating a healthy intestinal microbiota with prebiotics and probiotics can be tried as an alternative treatment for such diseases. There are few studies in the literature examining the relationship between urticaria and the intestinal microbiome; therefore, more studies with more samples are needed to prove the effect of urticaria and *Blastocystis* spp. on the microbiota.

## Figures and Tables

**Table 1:** Comparison of alpha diversity indices (Shannon, Simpson, and Chao-1) between study groups.

	Study Groups		
	Healthy Control (*n* = 34)	Urticaria (*n* = 33)	*p*-Value
Shannon entropy index			0.740 ^1^
Mean ± SD (range)	3.98 ± 1.33 (1.26–5.98)	4.07 ± 0.91 (2.44–5.73)	
Median (IQR)	4.30 (3.22–5.06)	4.23 (3.27–4.65)	
Simpson index			0.955 ^2^
Mean ± SD (range)	0.80 ± 0.19	0.83 ± 0.11	
Median (IQR)	0.86 (0.75–0.93)	0.84 (0.77–0.92)	
Chao-1 index			0.702 ^2^
Mean ± SD (range)	105.09 ± 80.53	79.34 ± 34.71	
Median (IQR)	77 (41–155.63)	71 (57–93)	

Values are presented as mean ± standard deviation (range: min–max) and median with interquartile range (IQR: 25th percentile–75th percentile). ^1^ Welch’s *t*-test. ^2^ Mann–Whitney *U* test.

**Table 2:** Comparison of alpha diversity indices (Shannon, Simpson, and Chao-1) according to the presence of *Blastocystis* spp.

	Study Groups		
	*Blastocystis*-Negative (*n* = 32)	*Blastocystis*-Positive (*n* = 35)	*p*-Value
Shannon entropy index			0.408 ^1^
Mean ± SD (range)	3.91 ± 1.18 (1.62–5.83)	4.14 ± 1.09 (1.26–5.98)	
Median (IQR)	4.09 (2.86–4.92)	4.39 (3.35–4.78)	
Simpson index			0.191 ^2^
Mean ± SD (range)	0.80 ± 0.15 (0.42–0.97)	0.84 ± 0.15 (0.27–0.68)	
Median (IQR)	0.83 (0.70–0.91)	0.88 (0.82–0.92)	
Chao-1 index			0.444 ^2^
Mean ± SD (range)	86.10 ± 56.90 (21–228.5)	98.18 ± 68.78 (18–325.1)	
Median (IQR)	66.5 (49.5–98.25)	72 (57.5–108.5)	

Data were expressed as mean ± standard deviation (range: min–max) and median with interquartile range (IQR: 25th percentile–75th percentile). ^1^ Studnet’s *t*-test. ^2^ Mann–Whitney *U* test.

**Table 3:** Microbiota differences between the urticaria patient group and the control group.

Bacteria	Statistic	*p*-Value
Firmicutes	536.5	0.763
Proteobacteria	355	0.015
Bacteroidetes	290	0.008
Faecalibacterium	446	0.151
Escherichia	311.5	0.005
Succinivibrionaceae	92.5	0.099
Phocaelcola	236	0.043
Diallister	317.5	0.557
Provetella	173.5	0.047
Roseburia	374.5	0.973
Veilonella	106.5	0.901
FtoBratio	321	0.026

FTOBratio: Comparison of the entire microbiota between two groups.

**Table 4:** Comparison of intestinal microbiota composition between *Blastocystis* spp.-positive and -negative samples.

Bacteria	Statistic	*p* Value
Firmicutes	452.5	0.179
Proteobacteria	517	0.748
Bacteroidetes	263	0.003
Faecalibacterium	435.5	0.120
Escherichia	495.5	0.684
Succinivibrionaceae	115	0.348
Phocaelcola	227.5	0.032
Diallister	335	0.823
Provetella	179.5	0.064
Roseburia	363	0.807
Veilonella	82.5	0.143
FtoBratio	292	0.009

FTOBratio: Comparison of the entire microbiota between two groups.

## Data Availability

All data generated or analyzed during this study are included in this published article.

## References

[1] Kocatürk Göncü E., Aktan Ş., Atakan N., Bülbül Başkan E., Erdem T., Koca R., Şavk E., Taşkapan O., Utaş S. (2016). Türkiye ürtiker tanı ve tedavi kılavuzu-2016. Turkderm-Arch. Turk. Dermatol. Venerol..

[2] Korkmaz Adiyaman G., Al Doğruman F., Mumcuoğlu I. (2015). Dışkı örneklerinde *Blastocystis* spp. varlığının mikroskobik, kültür ve moleküler yöntemlerle araştırılması. Mikrobiyoloji Bülteni.

[3] Jiménez P.A., Jaimes J.E., Ramírez J.D. (2019). A summary of *Blastocystis* subtypes in North and South America. Parasites Vectors.

[4] Duan J., Liu C., Bai X., Zhao X., Jiang T. (2023). Global trends and hotspots of gastrointestinal microbiome and toxicity based on bibliometrics. Front. Microbiol..

[5] Lazar V., Ditu L.M., Pircalabioru G.G., Gheorghe I., Curutiu C., Holban A.M., Picu A., Petcu L. (2018). Aspects of gut microbiota and immune system interactions in infectious diseases, immunopathology, and cancer. Front. Immunol..

[6] Chen Y., Wang X., Zhang C., Liu Z., Li C., Ren Z. (2022). Gut microbiota and bone diseases: A growing partnership. Front. Microbiol..

[7] Engevik M.A., Luk B., Chang-Graham A.L., Hall A., Herrmann B., Ruan W., Endres B.T., Shi Z., Garey K.W., Hyser J.M. (2019). *Bifidobacterium dentium* fortifies the intestinal mucus layer via autophagy and calcium signaling pathways. mBio.

[8] Goto Y. (2019). Epithelial cells as a transmitter of signals from commensal bacteria and host immune cells. Front. Immunol..

[9] Paone P., Cani P.D. (2020). Mucus barrier, mucins and gut microbiota: The expected slimy partners?. Gut.

[10] Kunst C., Schmid S., Michalski M., Tümen D., Buttenschön J., Müller M., Gülow K. (2023). The influence of gut microbiota on oxidative stress and the immune system. Biomedicines.

[11] Chu J., Feng S., Guo C., Xue B., He K., Li L. (2023). Immunological mechanisms of inflammatory diseases caused by gut microbiota dysbiosis: A review. Biomed. Pharmacother..

[12] Maciel-Fiuza M.F., Muller G.C., Campos D.M.S., do Socorro Silva Costa P., Peruzzo J., Bonamigo R.R., Veit T., Viana F.S.L. (2023). Role of gut microbiota in infectious and inflammatory diseases. Front. Microbiol..

[13] Valsecchi R., Leghissa P., Greco V. (2004). Cutaneous lesions in Blastocystis hominis infection. Acta Derm.-Venereol..

[14] Lim M.X., Png C.W., Tay C.Y.B., Teo J.D.W., Jiao H., Lehming N., Tan K.S.T., Zhang Y. (2014). Differential regulation of proinflammatory cytokine expression by mitogen-activated protein kinases in macrophages in response to intestinal parasite infection. Infect. Immun..

[15] Ragavan N.D., Kumar S., Chye T.T., Mahadeva S., Shiaw-Hooi H. (2015). *Blastocystis* sp. in irritable bowel syndrome (IBS)-detection in stool aspirates during colonoscopy. PLoS ONE.

[16] Uranga J.A., Martínez V., Abalo R. (2020). Mast cell regulation and irritable bowel syndrome: Effects of food components with potential nutraceutical use. Molecules.

[17] Aydemir S., Arvas Y.E., Aydemir M.E., Barlık F., Gürbüz E., Yazgan Y., Ekici A. (2025). Antiprotozoal Effects of Pediococcus acidilactici-Derived Postbiotic on Blastocystis Subtypes ST1/ST3. Pathogens.

[18] Nabizadeh E., Jazani N.H., Bagheri M., Shahabi S. (2017). Association of altered gut microbiota composition with chronic urticaria. Ann. Allergy Asthma Immunol..

[19] Fasano A. (2020). All disease begins in the (leaky) gut: Role of zonulin-mediated gut permeability in the pathogenesis of some chronic inflammatory diseases. F1000Research.

[20] Gurung M., Li Z., You H., Rodrigues R., Jump D.B., Morgun A., Shulzhenko N. (2020). Role of gut microbiota in type 2 diabetes pathophysiology. eBioMedicine.

[21] Fan Y., Pedersen O. (2021). Gut microbiota in human metabolic health and disease. Nat. Rev. Microbiol..

[22] Mariat D., Firmesse O., Levenez F., Guimarăes V., Sokol H., Doré J., Furet J.P. (2009). The *Firmicutes*/*Bacteroidetes* ratio of the human microbiota changes with age. BMC Microbiol..

[23] Ismail N.A., Ragab S.H., Abd ElBaky A., Shoeib A.R., Alhosary Y., Fekry D. (2011). Frequency of Firmicutes and Bacteroidetes in gut microbiota in obese and normal weight Egyptian children and adults. Arch. Med. Sci..

[24] Koliada A., Syzenko G., Moseiko V., Budovska L., Puchkov K., Perederiy V., Gavalko Y., Dorofeyev A., Romanenko M., Tkach S. (2017). Association between body mass index and Firmicutes/Bacteroidetes ratio in an adult Ukrainian population. BMC Microbiol..

[25] Schwiertz A., Taras D., Schäfer K., Beijer S., Bos N.A., Donus C., Hardt P.D. (2010). Microbiota and SCFA in lean and overweight healthy subjects. Obesity.

[26] Muñoz-Yáñez C., Méndez-Hernández A., González-Galarza F.F., Prieto-Hinojosa A.I., Guangorena-Gómez J.O. (2025). Diet Quality Modulates Gut Microbiota Structure in Blastocystis-Colonised Individuals from Two Distinct Cohorts with Contrasting Sociodemographic Profiles. Microorganisms.

[27] Yason J.A., Liang Y.R., Png C.W., Zhang Y., Tan K.S.W. (2019). Interactions between a pathogenic *Blastocystis* subtype and gut microbiota: In vitro and in vivo studies. Microbiome.

[28] Yañez C.M., Hernández A.M., Sandoval A.M., Dominguez M.A.M., Muniz S.A.Z., Gomes J.A.G. (2021). Prevalence of *Blastocystis* and its association with *Firmicutes/Bacteroidetes* ratio in clinically healthy and metabolically ill subjects. BMC Microbiol..

